# On the Existence of Step-To-Step Breakpoint Transitions in Accelerated Sprinting

**DOI:** 10.1371/journal.pone.0159701

**Published:** 2016-07-28

**Authors:** Gertjan Ettema, David McGhie, Jørgen Danielsen, Øyvind Sandbakk, Thomas Haugen

**Affiliations:** 1 Centre for Elite Sports Research, Department of Neuroscience, Faculty of Medicine, Norwegian University of Science and Technology, Trondheim, Norway; 2 Norwegian Olympic Federation, Oslo, Norway; Universite de Nantes, FRANCE

## Abstract

Accelerated running is characterised by a continuous change of kinematics from one step to the next. It has been argued that breakpoints in the step-to-step transitions may occur, and that these breakpoints are an essential characteristic of dynamics during accelerated running. We examined this notion by comparing a continuous exponential curve fit (indicating continuity, i.e., smooth transitions) with linear piecewise fitting (indicating breakpoint). We recorded the kinematics of 24 well trained sprinters during a 25 m sprint run with start from competition starting blocks. Kinematic data were collected for 24 anatomical landmarks in 3D, and the location of centre of mass (CoM) was calculated from this data set. The step-to-step development of seven variables (four related to CoM position, and ground contact time, aerial time and step length) were analysed by curve fitting. In most individual sprints (in total, 41 sprints were successfully recorded) no breakpoints were identified for the variables investigated. However, for the mean results (i.e., the mean curve for all athletes) breakpoints were identified for the development of vertical CoM position, angle of acceleration and distance between support surface and CoM. It must be noted that for these variables the exponential fit showed high correlations (r^2^>0.99). No relationship was found between the occurrences of breakpoints for different variables as investigated using odds ratios (Mantel-Haenszel Chi-square statistic). It is concluded that although breakpoints regularly appear during accelerated running, these are not the rule and thereby unlikely a fundamental characteristic, but more likely an expression of imperfection of performance.

## Introduction

Linear sprint running, including acceleration and maximal sprint velocity, has received considerable attention in research literature. Recently, Slawinski et al. [1] showed that the maximal sprint velocity and mean power output (W·kg-1) developed over the entire distance strongly influenced 100-m performance in male and female world-class sprinters. Team sport athletes typically achieve peak velocity after 30–40 m of maximal sprinting [2, 3], while the world’s fastest male and female 100-m performers are able to accelerate with maximal intensity for 50–80 m, reaching maximal velocity values >12 and 11 m·s^-1^, respectively [1, 4]. The mathematical behaviour of the acceleration of the body centre of mass (CoM) from zero towards maximal velocity can be described by an exponential function [5, 6].

Accelerated running and maximal velocity sprinting can be considered as two running modes with different characteristics according to body configuration (forward leaning trunk in accelerated running versus upright posture at maximal velocity), CoM positioning relative to support base and step characteristics such as step length, contact time and aerial time [7–11]. On the basis of these differences between accelerated running and maximal velocity sprinting, a further division of the acceleration phase has been proposed (e.g., [8–10]). Such division, however, often seems relatively arbitrary and is typically based on differences in the so-called state-space, i.e., position and velocity of body segments rather than clearly identifiable points in time where detectable changes occur. As an exception, Nagahara et al. [10] analysed changes in step characteristics in more depth and identified three phases of accelerated running based on changes in the relationship between acceleration and rates of change of step frequency and step length. Thus, the rationale of dividing accelerated running in subsections has been extended in that various abrupt changes (i.e., breakpoints) may occur that define the phases during accelerated running distinctly [9, 10]. As opposed to this idea, the notion that sprint velocity is well described by an exponential function suggests that, at least with regard to performance outcome, sprint running is guided by a continuous change-over from start to maximal sprint velocity. Whether breakpoints occur on a regular basis or only occasionally in accelerated running is both of theoretical and practical relevance; the answer to this question provides information about whether essential differences exist between the different phases of sprint running or if these phases are a mere artificial division. From a practical perspective, the interpretation of any breakpoint in an individual run depends on if the occurrence is the rule, i.e., guided by mechanical principles, or if it is an exception, possibly indicating an imperfection in the performance.

Although breakpoints may exist, the empirical evidence so far is limited. For example, Nagahara et al. [10] applied a linear curve fitting procedure that allowed the emergence of two breakpoints for the vertical position of CoM (S_*vCoM*_) against time. However, they did not test the goodness-of-fit against a fitting procedure that assumes a continuous change (i.e., an exponential curve). Thus, Nagahara et al. [10] did not present statistical evidence about the existence of breakpoints, because their method a priori assumes that such breakpoints exist. However, their data suggest that, at least in individual cases and at face value, breakpoints in the increase of CoM height indeed exist, as is also indicated previously [12, 13].

Nagahara et al. [10] argued that S_*vCoM*_ is a logical variable to study potential breakpoints, but other factors such as step length, contact time and aerial time are also likely candidates for this purpose.

**Fig 1:**
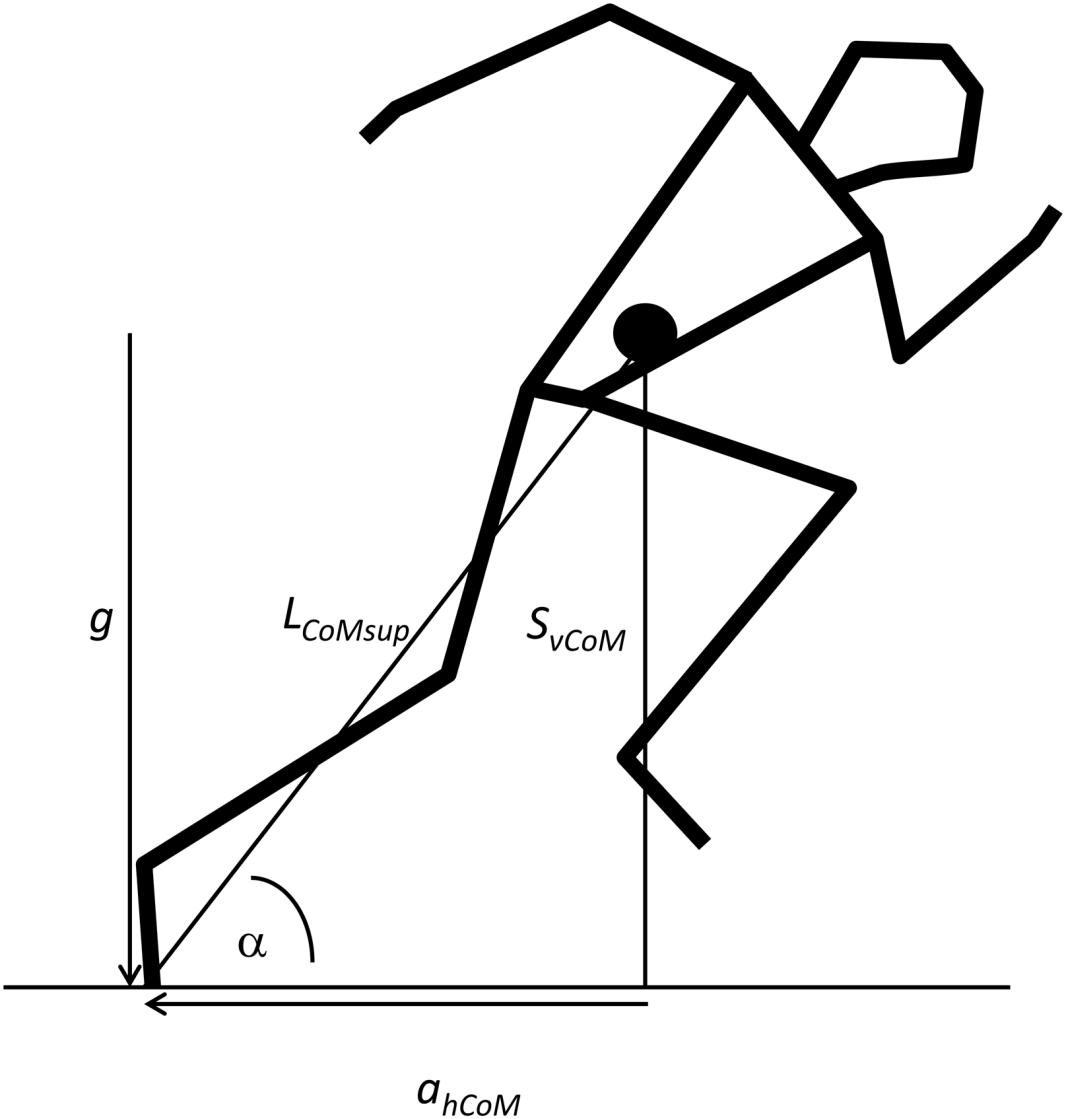
Schematic drawing of a position during accelerated running. The variables described in eqs 1–3 and their interrelationships are graphically presented. Accelerations *g* and *a*_*hCoM*_ are defined as by di Prampero et al. [6]. Solid circle is CoM.

From a theoretical point of view, S_*vCoM*_ is mechanically closely related to acceleration (Fig 1); di Prampero et al. [6] describe how the angle of the line from CoM to the surface support point with the horizontal theoretically depends on forward acceleration, and thus can be referred to as the angle of acceleration (α_*accCoM*_):

$$\alpha_{accCoM=\text{atan}\left(\frac{g}{a_{hCoM}}\right)}$$ (1)

*g* representing the gravitational acceleration and *a*_*hCoM*_ the horizontal acceleration. The angle of acceleration and height of CoM are mechanically linked according (eq 2)

$$\alpha_{accCoM=\text{asin}\left(\frac{S_{vCoM}}{L_{CoMsup}}\right)}$$ (2)

L_*CoMsup*_ is the distance between CoM and the surface support point (i.e., forefoot in sprint running). From eqs 1 and 2 it follows that

$$S_{vCoM}=\frac{L_{CoMsup\;\times \;}g}{\sqrt{a_{hCoM}^{2}+g^{2}}}$$ (3)

Thus, S_*vCoM*_ is directly related to horizontal acceleration and thereby to the development of horizontal velocity (i.e., running speed). In other words, if velocity would develop in a smooth exponential way, it would be rather surprising if S_*vCoM*_ shows breakpoints. Of course, the possibility exists that both body configuration, expressed in L_*CoMsup*_, and S_*vCoM*_ show breakpoints that together lead to a smooth development of acceleration angle.

The aim of the present study was therefore, in the framework of theory development on accelerated sprinting, to test whether breakpoints in S_*vCoM*_, associated variables and more common step variables exist. This was done by comparing two curve-fitting approaches on the relevant kinematical variables: an exponential fit was used on the basis of the assumption of continuity and a piecewise linear-exponential on the assumption of the existence of (mathematically discontinuous) breakpoints. Horizontal velocity (V_*hCoM*_), S_*vCoM*_, angle of acceleration and L_*CoMsup*_ were analysed. In addition, well studied variables such as contact time, aerial time and step length were also considered.

## Methods

### Participants

Twenty-four male Norwegian competitive sprinters (age 23.1 ± 3.4 yr, height 1.81 ± 0.06 m, body mass 75.5 ± 5.5 kg, lean body mass 70.4 ± 4.8 kg, fat mass 9.7 ± 1.4%, personal best 100 m 10.86 ± 0.22 s) voluntarily signed up for this study. All athletes were healthy and free of injuries at the time of testing, and the study was approved by The Norwegian Data Protection Authority. All participants signed an informed consent form before the experiment and were made aware that they could withdraw from the study at any point without providing an explanation. The study was conducted in accordance with the Declaration of Helsinki.

### Protocol

All experiments were performed over a period of three days on a competition indoor running track. The middle of the straight was used for data collection. All sprints were performed from starting blocks according to the competition rules outlined by the International Athletic Association Federation [14]. A finish line was marked 25 m from the start (a larger distance was not feasible because of limited length of the straight in the hall where recordings were possible). Athletes were instructed to perform a sprint run as in regular competition, maintaining maximal effort until they passed the 25 m finish line. The actual data recording was done from starting blocks to 18 m (limited by the number of cameras, but sufficient to study the existence of the first breakpoint). After a self-selected warm-up procedure the athletes performed two or three maximal accelerated sprint starts separated by ample recovery time. During this data collection, a similar number of maximal velocity runs were recorded (with free in-run to come at maximal speed at the recording area). These data are not relevant for the current analysis and not reported. However, this procedure limited the number of repetitions that could be executed with full effort and without risk of injury. Prior to each run, the athletes indicated if they were ready for a maximal effort. A sprint start was considered successful if the athlete indicated directly after the exercise that he was satisfied with his performance.

### Measurements

Kinematics were recorded in 3D using the Qualisys motion tracking system with 21 Oqus cameras (Qualisys AB, Gothenburg, Sweden) at a 250 Hz sample rate. The cameras were placed at both sides of the running track and the volume of measurement was calibrated according to the manufacturer’s specifications. The resolution of marker position was <2 mm. Care was taken that the x-axis of the coordinate system was in the running direction. Reflective markers (⌀ 19 mm) were placed at anatomical landmarks to identify 12 segments and related joint movements of the body: forehead and C7, bilaterally on the lateral malleolus, lateral femoral epicondyle, trochanter major, anterior superior iliac spine, posterior superior iliac spine, lateral tip of the acromion, lateral humeral epicondyle, ulnar styloid process and on both shoes: heel, hallux and above the head of the 5^th^ metatarsal. All data were recorded using the QTM software v2.12 (Qualisys AB, Gothenburg, Sweden) and all post-analysis was performed in Matlab R2014a (The Mathworks Inc.). Marker position data were filtered using a Chebyshev Type II low pass filter (cut-off 20Hz, 16^th^ order). Position of CoM was calculated according to de Leva [15]. Touch-down and lift-off of the foot was determined by a purpose-written algorithm based on Nagahara et al. [10]. First, the approximate epoch for each touch-down and lift-off was found by identifying the time that the height of the 5^th^ metatarsal marker on each foot decreased to under and increased above a set threshold (40 mm), respectively. Thus, first an epoch around and slightly exceeding the actual ground contact was determined. Within this epoch, the first and last peak vertical acceleration of the metatarsal marker was used to indicate the exact time of touch-down and lift-off, respectively. In this way, contact and aerial phases for each step could be identified and variable values related to these periods calculated.

Velocity and acceleration of markers and calculated variables (e.g., CoM) were derived by applying numerical differentiation of the position signals. CoM’s horizontal velocity and acceleration were used as running velocity and acceleration, respectively.

### Curve fitting and statistical procedure

The average vertical position of CoM (i.e., S_*vCoM*_) and mean horizontal velocity (i.e., V_*hCoM*_) during ground contact were used as the main variables for identification of breakpoints in kinematics and performance outcome, respectively. Exponential (eq 4) and piecewise (eqs 5a and 5b) functions were fitted through these data against time. Two different piecewise functions were used; one consisting of two discontinuous linear parts (eq 5a) and one of a linear and exponential part (eq 5b). The piecewise functions have a larger number of degrees of freedom and therefore should provide a better goodness-of-fit than the exponential fit. This improvement of goodness-of-fit by applying the piecewise functions was tested for significance using the F-test on differences in residual sum of squares. Note that only the best result of the two piecewise options was used in this statistical analysis. Additionally, contact time, aerial time and step length were treated in the same manner. Step frequency was not included for this analysis because this variable is close to maximal from the first step and hardly changes afterwards, e.g., [8–10].

Because the time line has an arbitrary start (i.e., t = 0) with respect to the time moment that the first full step occurred, the curve fitting equations allowed for a time offset, but this offset constant was not considered in determination of the degrees of freedom. The equations without the time-offset constant are:

$$y=a\left(1\pm e^{\left(-\frac{t}{b}\right)}\right)$$ (4)

$$y=\{\begin{array}{rr} at, & t<k\\ \;b+ct, & t\geq k \end{array}$$ (5a)

$$y=\{\begin{array}{rr} at, & t<k\\ c\left(1\pm e^{\left(-\frac{t}{b}\right)}\right), & t\geq k \end{array}$$ (5b)

with *y* the variable of interest *a*, *b* and *c* the fitting constants and *k* the time of transition between the first and second piece of the piecewise function. Fitting constants and *k* were found by the fitting iteration. All fitting procedures were performed in MatlabR2014a (Mathworks, Natick, Massachusetts, USA), using its *fit* function. The original data are found in supporting data S1 File.

In order to avoid discontinuities that may be caused by different conditions created by standing in the starting blocks and having ground contact with the hands, only data from complete ground contact periods, i.e., initiated by a touch-down event and finished by a lift-off event, were used. Thus, the lift-offs out of the starting blocks were not considered in this analysis.

**Fig 2:**
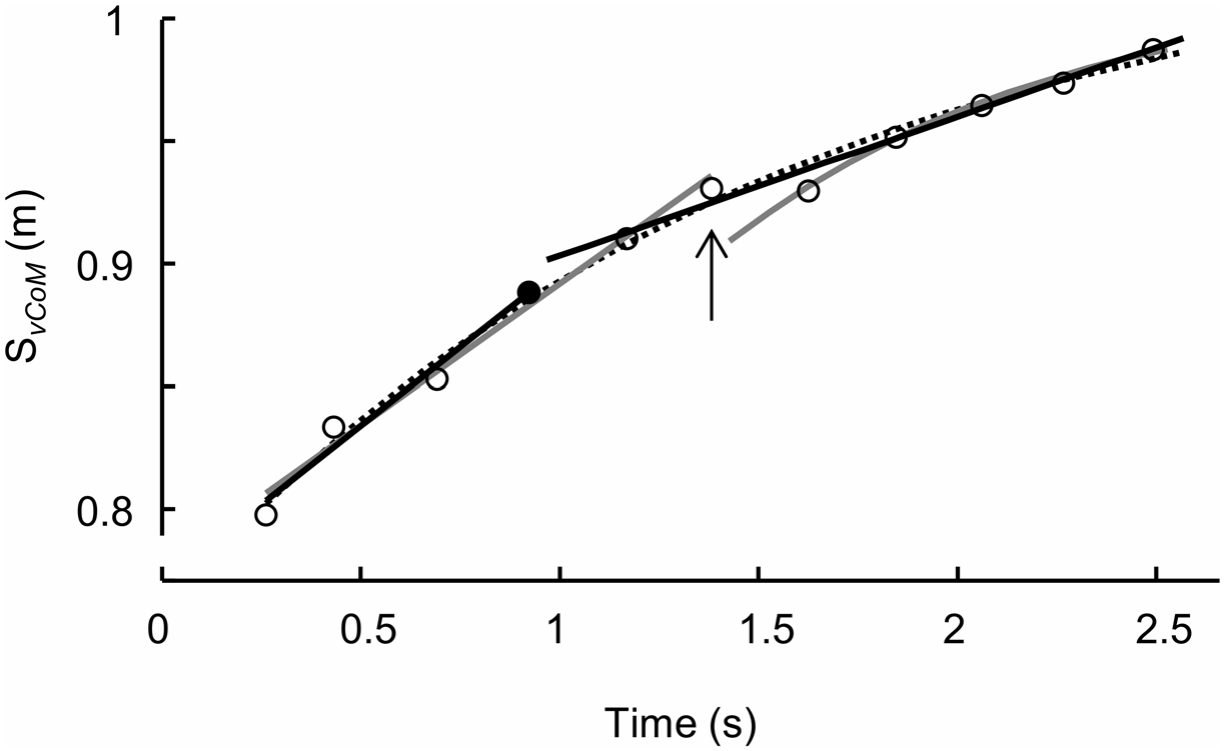
Example of CoM’s vertical position and curve fitting from step to step in time. Open markers are data, solid marker indicates the breakpoint according to Nagahara et al. [10] and piecewise twice linear fit (solid lines, Eq 5a). Vertical arrow indicates the breakpoint according to linear-exponential piecewise fit (grey curves, Eq 5b). Dotted line is exponential fit (Eq 4).

Using this procedure, we re-examined the notion of the existence of breakpoints [10]. There, the first breakpoint was indicated to occur at around step 3–6. The procedure applied by Nagahara et al. [10] to identify the first breakpoint step was also employed here and presented in Fig 2 for comparison reasons. The second suggested breakpoint at about step 10–18 [10] was not considered in this study because our data included at the most 13 steps.

To test if occasions of breakpoints for S*v*_*CoM*_, α_*accCoM*_ and L_*CoM*_ were related, odds ratios for pairs of variables for all runs were tested using the Mantel-Haenszel Chi-square statistic.

## Results

Forty-one sprint starts were recorded successfully for the current analysis. The average 20 m sprint times were 3.06 ± 0.06 s. Fig 2 shows an example of a fit for the mean CoM vertical position during ground contact. Even though visual inspection shows a potential breakpoint between step 6 and 7, all fitting procedures show a good fit with the experimental data (r^2^>0.99). Note that the piecewise twice linear fit would have indicated a breakpoint between step 4 and 5 (as would Nagahara et al.’s procedure [10]), not between 6 and 7 (piecewise linear-exponential).

Table 1 shows an overview of the comparison of fitting procedures for the analysed variables. About half of the runs (19 out of 41) showed a significant improvement of the fit for S_*vCoM*_ by using a piecewise function (twice linear or linear–exponential versus a pure exponential curve). Three of the 41 runs showed an improvement of the fit on horizontal velocity, but only if the piecewise linear-exponential combination was used. It should be noted that the exponential function fitted the velocity data extremely well (r^2^>0.993) and the S_*vCoM*_ data well (r^2^>0.865). The other variables showed a better piecewise fit in about 10 runs. In 16 runs, L_*CoMsup*_ did not show a very clear pattern (either linear or exponential), and these were excluded from further analysis with regard to this variable. Thus only 25 of 41 runs were analysed for this variable (see also discussion). For contact time, this occurred in 3 runs.

**Table 1:** Comparison of piecewise linear/exponential and exponential curve fitting.

	S_*vCoM*_		α_*accCoM*_		L_*CoMsup*_		V_*hCoM*_		Contact Time		Aerial Time		Step Length	
Runs	19	(41)	11	(41)	12	(25)	3	(41)	12	(38)	10	(41)	9	(41)
P (on mean)	0.020		0.030		0.024		1		0.055		0.073		0.566	
r^2^ (on mean)	0.993		0.991		0.941		0.999		0.994		0.991		0.997	

Runs: number of all runs (total in parentheses) with valid exponential fit and where piecewise fit performs better (p<0.05), P: the p-value for improvement of the best piecewise fit (eqs 5a and 5b) in comparison to the exponential fit (eq 4) for the mean data over all athletes. r^2^: the r^2^ value for the exponential fit for the mean data over all athletes.

**Fig 3:**
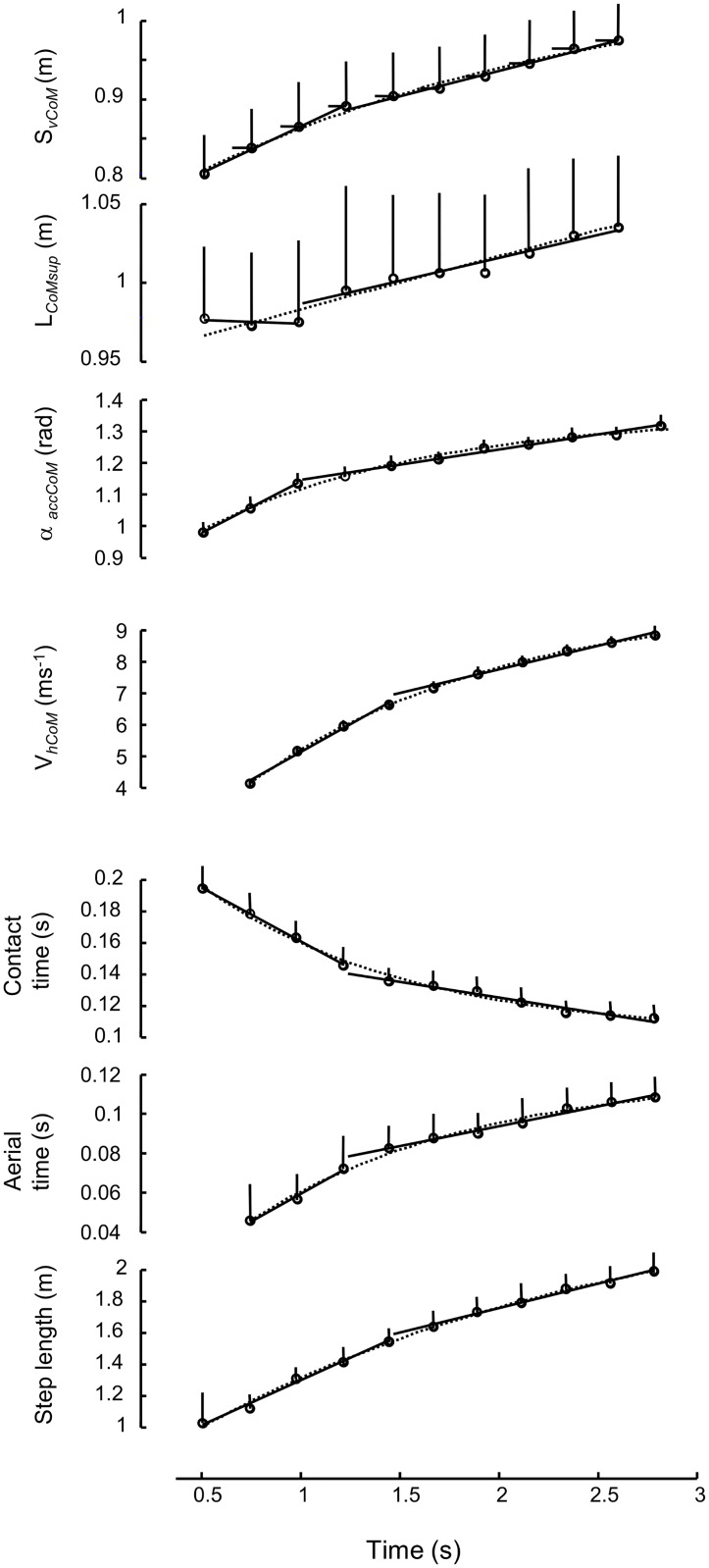
Curve fittings for the mean data for each step over all athletes. Only steps with a complete data set for the variable at hand are shown and were used for the fitting procedures. Dotted line: exponential; Solid lines: piecewise linear. Horizontal (top diagram only) and vertical bars are standard deviation (n = 24). Note that the seemingly very low standard deviation, especially for horizontal velocity, is only partly due to the homogeneous group and mainly due to scaling of the diagram, which covers low velocity at the first steps to almost maximal sprinting velocity. Time = 0 is the time of the first movement of CoM during the sprint start.

The mean curves over all 24 athletes were based on a weighted average of all successful runs. That is, for the sixteen athletes who performed more than one successfully recorded sprint (two in 15 cases; three in one case), these multiple runs were first averaged for each athlete before these averages were used for finding the grand average over all athletes. When considering the mean curves over all athletes (Fig 3), it should be noted that the exponential functions fit all average data extremely well (as indicated by the r^2^ values). Still, the development of S_vCoM_, α_*accCoM*_ and L_*CoMsup*_ showed a significant improvement using the piecewise function (Table 1, p-values). Note that horizontal velocity development showed no such improvement. In fact, for V_*hCoM*_ the exponential curve showed an almost perfect fit which was better than the piecewise fit, despite the higher degrees of freedom (Table 1). Contact time, aerial time and step frequency show no improvement by using piecewise functions.

The Mantel-Haenszel’s statistic for odds ratios showed no significant relationship between variables (Table 2). In other words, no evidence was found for a relationship between occurrence of breakpoints for S*v*_*CoM*_, α_*accCoM*_ and L_*CoM*_.

**Table 2:** Odds ratios for incidences of breakpoints (Pos) or continuity (Neg) as indicated by statistical comparison of the piecewise function (eqs 5a and 5b) and the exponential function (eq 4) for S*v*_*CoM*_, α_*accCoM*_ and L_*CoMsup*_.

**A**				
	P = 0.945	α_*accCoM*_		
		Pos	Neg	Total
S*v*_*CoM*_	Pos	5	14	19
	Neg	6	16	22
	Total	11	30	41
**B**				
	P = 0.851	L_*CoMsup*_		
		Pos	Neg	Total
S*v*_*CoM*_	Pos	6	6	12
	Neg	6	7	13
	Total	12	13	25
**C**				
	P = 0.144	L_*CoMsup*_		
		Pos	Neg	Total
α_*accCoM*_	Pos	5	2	7
	Neg	7	11	18
	Total	12	13	25

The odds ratios were calculated only for those runs in which both variables showed reasonable to good fit for both functions. L_*CoMsup*_ was only fitted satisfactorily in 25. Tables A, B and, C compare two of the three variables pairwise. Pos indicates the existence of a breakpoint; Neg indicates continuity. P-values are for the Mantel-Haenszel Chi-square statistic, which are all well above 0.05, indicating that the occurrence of breakpoints do not coincide in a systematic way among the variables investigated.

## Discussion

The aim of this study was to examine if step-to-step breakpoints in the transitions of kinematics during accelerated running occur. Our results only partially confirm the existence of breakpoints suggested by Nagahara et al. [10]. In general, the exponential fits showed very high correlation values, indicating that transitions are generally smooth. However, the changes of some kinematic variables tested here were still fitted better by a piecewise function, both in individual runs and for the mean curves. These findings indicate that subtle breakpoint transitions occasionally occur. This is strengthened by the breakpoints found in the overall mean curves, because the smoothing effect of the averaging procedure favours the smooth outcome without breakpoints. Still, for each variable, the majority of individual runs show no breakpoints. Basically no breakpoints at all are found in velocity development (three out of 41 when using linear-exponential piecewise fit of data already extremely well fitted by the exponential function).

Maybe the most important question is if the observed breakpoints are to be considered a fundamental characteristic for accelerated running. Our results suggest that this is not likely because the majority of runs do not show breakpoints. Thus, other reasons, e.g., imperfections of performance or possibly a mere bilateral asymmetry (which seems to be more likely norm than exception, e.g., [16–18]) should be considered. Our results and interpretation are in contradiction with Nagahara et al. [10] who, by choice of analysis method, a priori assumed that breakpoints occur. Still, the mean data (Table 1, Fig 3) show breakpoints for all three variables that are directly related to the elevation of CoM (S*v*_*CoM*_, α_*accCoM*_, L_*CoM*_; eqs 1–3). Thus, the breakpoints that do seem to occur must be of such magnitude that they affect the mean curves significantly. Given this statistical impact, we refute the notion that these occurrences are merely caused by noise in the data. Ideally, we would have recorded more sprints for each athlete, so that each athlete’s average curves could have been analysed as a better representative individual curve. By analysing both at the level of each individual run as well as the grand average for all athletes, we have tried to minimise the chance that mere noise would have affected the outcome. Hence, we conclude that breakpoints occur but cannot be an essential characteristic of accelerated running. Considering that all analysed runs are regarded as good performance, a breakpoint cannot be regarded as a “failure of performance” either. Thus, we would conclude, as opposed to Nagahara et al. [10], that from a practical point of view, the occurrence of a breakpoint in an athlete’s performance may be cause for some concern about the technical execution of such effort. However, this study does not suffice to make a final verdict about the implication of breakpoints in accelerated running.

Interestingly, even though quite some runs with breakpoints on the fundamental variables for kinematic development (S*v*_*CoM*_, α_*accCoM*_, L_*CoM*_) were found, running speed itself always seems to develop in a perfect exponential manner. This may strengthen the notion that breakpoints are not a key characteristic in accelerated running. These three variables are related to each other and the development of running speed (i.e., a_*hCoM*_) by rules of mechanics (eqs 1–3). One might therefore speculate that any subtle disturbance in the execution of accelerated running that leads to breakpoints in one of the variables automatically causes disturbances to one or more of the other variables. Given the outcome of the chi-square statistics on the odds ratios, we did not find any indication of such a relationship, i.e., there was no obvious pattern for the incidences of breakpoints between the three kinematical variables (S*v*_*CoM*_, α_*accCoM*_, L_*CoM*_).

The L_*CoMsup*_ variable did not always show a clear piecewise or exponential pattern, and seemed more variable among runs and athletes regarding its development from step to step. We introduced this variable as the link between S*v*_*CoM*_ and α_*accCoM*_, the variables introduced by di Prampero et al. [6] and Nagahara et al. [10] (eqs 1–3). Its behaviour regarding development in time seems irregular (Fig 3), but this may be misleading: the change in L_*CoMsup*_ amounts only to about 5% of the mean value, much less than changes in α_*accCoM*_, S*v*_*CoM*_ and especially V_*hCoM*_. Also, the other three variables show a far larger change. In hindsight it is not surprising that the average distance between CoM and support area does not increase much from step to step during accelerated running. After all, it is mostly affected by the extension of the lower extremity, which must be close to maximal to obtain propulsion.

In conclusion, the present study identified the occurrences of step-to-step breakpoint transitions in accelerated running only in a minority of runs. No breakpoints were identified for performance outcome (i.e., running speed), which shows a near perfect exponential development. Therefore, it seems most likely that the occurrence of breakpoints is due to imperfections of locomotion performance rather than a fundamental characteristic for accelerated running.

## Supporting Information

S1 File Excel File containing all step data that the curve fitting analysis is based on. (XLSX)
